# Metastatic Neuroendocrine Carcinoma Presenting with Bilateral Axillary Lymphadenopathy

**DOI:** 10.7759/cureus.7575

**Published:** 2020-04-07

**Authors:** Hyunjoo Ko, Lynsey M Maciolek, Suimin Qiu, Linden Dixon, Quan D Nguyen

**Affiliations:** 1 Radiology, University of Texas Medical Branch, Galveston, USA; 2 Pathology, University of Texas Medical Branch, Galveston, USA

**Keywords:** small cell lung cancer, lung cancer, breast cancer, bilateral breast masses, metastastic high-grade neuroendocrine carcinomas, metastasis, lung masses in smokers, small blue cells, hypoechoic axillary masses, primary breast malignancy

## Abstract

Metastatic, high-grade neuroendocrine carcinomas are frequently associated with small cell lung cancer (SCLC), classically spreading to the liver, bone, lung, and brain. Though SCLCs most commonly present as large masses interfering with the airway, this malignancy may appear initially as a benign mass at a distant site. This case profiles a 64-year-old woman who presented with bilateral breast masses that were identified as metastases of poorly differentiated, high-grade neuroendocrine SCLC through mammogram, ultrasound, CT, and core biopsy. Accurately identifying etiology of a breast malignancy is critical to therapeutic planning, as disparate treatment guidelines and disease courses exist for primary breast cancer and SCLC.

## Introduction

Small cell lung carcinoma (SCLC) is a high-grade neuroendocrine tumor marked by rapid cell growth and early metastases to multiple organ systems. The underlying pathophysiology is hypothesized to stem from loss of functionality at multiple points along chromosome 3p as well as other genetic cell growth modulators, such as p53, RB1, and c-Kit [1]. Significant smoking history is virtually always present in patients, pointing to chronic smoking-related toxin exposure as a major risk factor for SCLC.

SCLC typically presents in heavy smokers with nonspecific obstructive respiratory symptoms as the initial malignancy develops and expands in the lumen of a central airway. After identifying a likely primary lesion on imaging, tissue biopsy and histology are necessary to distinguish SCLC from metastases or other primary lung malignancies. SCLC is classically characterized as small “blue” cells with large amounts of finely dispersed chromatin and scant cytoplasm. These cells grow rapidly in clusters or sheets, often with necrotic foci. Epithelial cell markers such as keratin and epithelial antigen are universal in small cell carcinoma; neuroendocrine markers such as synaptophysin and chromogranin are also very common, though not necessary for diagnosis [2]. Staging of SCLC is most commonly performed based on the Veterans’ Affairs Lung Study Group system, which divides patients with SCLC into two groups: limited disease, in which the tumor is localized to a single region of the thorax with limited lymph node involvement, and extensive disease, in which the tumor has metastasized to distant sites or caused neoplastic syndromes.

Any diagnosis of SCLC, including limited stage SCLC, warrants systemic therapy. Platinum therapy, etoposide, and an adjuvant immunomodulator comprise the backbone of initial induction therapy for SCLC, having demonstrated similar efficacy to other frequently used chemotherapy agents with fewer adverse effects [3,4]. Carboplatin-etoposide and atezolizumab is the most common therapeutic combination; carboplatin-etoposide and durvalumab is another immunochemotherapy that has demonstrated promise in recent trials [5].

## Case presentation

A 64-year-old woman presented to the clinic for a well-woman examination. Her past medical history includes hypothyroidism, skin squamous cell carcinoma, and atypical squamous cell changes of undetermined significance of the cervix. Notably, this patient has a 60 pack-year history of cigarette tobacco use.

**Figure 1:**
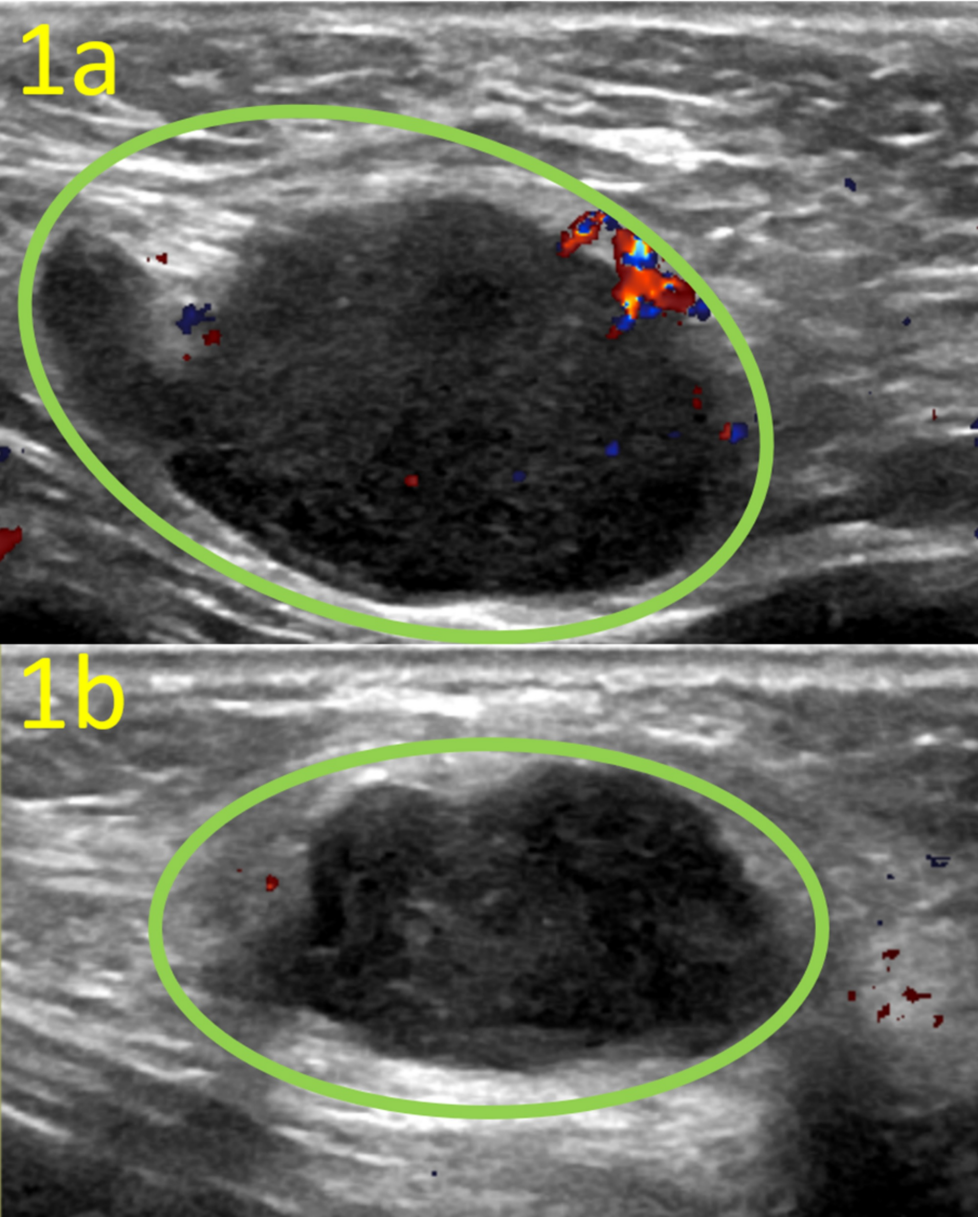
Targeted Ultrasound of the Bilateral Low Axilla These ultrasound images of the bilateral low axilla demonstrate morphologically abnormal lymph nodes (green circles) with loss of fatty hilum and cortical thickening. Left axillary lymph node (a) measures 35 x 19 x 31 mm; right axillary lymph node (b) measures 27 x 13 x 20 mm.

Routine physical exam revealed firm, mobile, nontender, and well-circumscribed bilateral axillary masses (3x2 cm left, 2x1 cm right) with corresponding adenopathy, and ultrasound and mammogram were ordered to evaluate potential breast malignancy. Initial diagnostic ultrasound revealed hypoechoic axillary masses associated with loss of fatty hilum, cortical thickening, and morphologically abnormal local lymph nodes categorized as Breast Imaging-Reporting and Data System Category 4A bilateral complex cysts (Figure 1).

**Figure 2:**
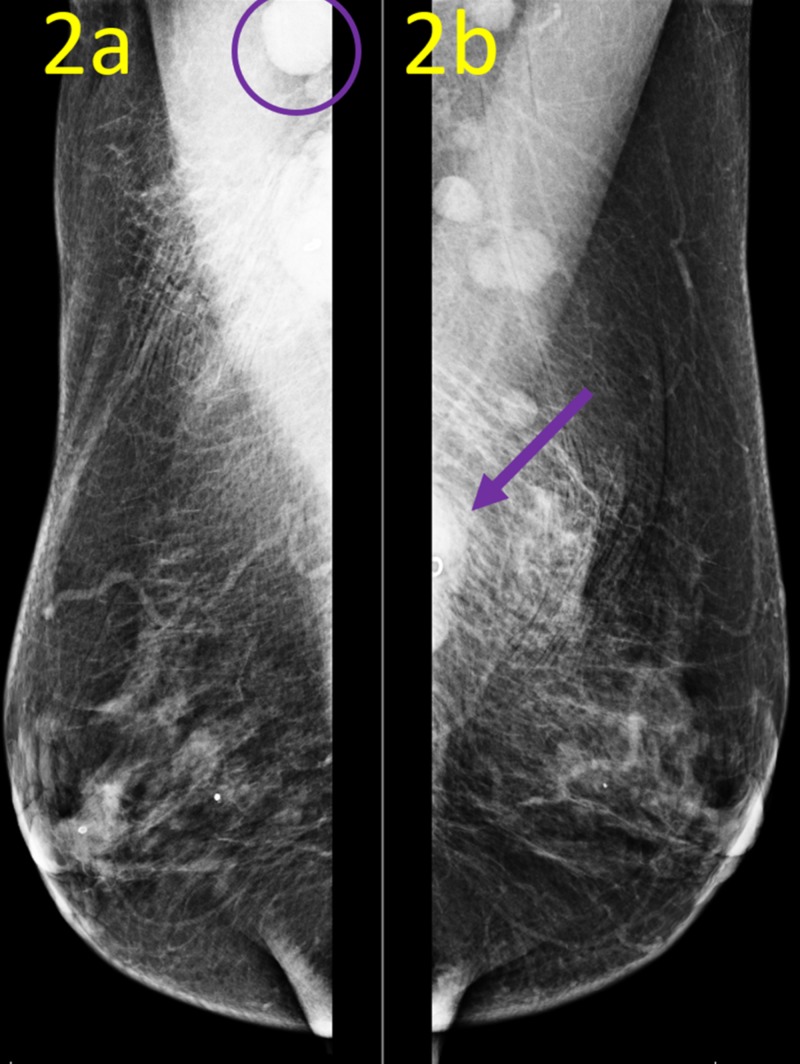
Postbiopsy Mammogram Mediolateral Oblique (MLO) Views These postbiopsy mammogram MLO views demonstrate biopsy markers: right axilla (a) and left axilla (b). Right biopsy marker clip is not included in the field of view, but a morphologically abnormal lymph node (purple circle) is seen in the high axilla. Left biopsy marker clip (purple arrow) seen in the left low axilla marks a morphologically abnormal lymph node.

Initial diagnostic mammogram did not visualize the lymph nodes and yielded no abnormal findings. Follow-up axillary mammogram confirmed ultrasound findings of morphologically abnormal lymph nodes (Figure 2).

**Figure 3:**
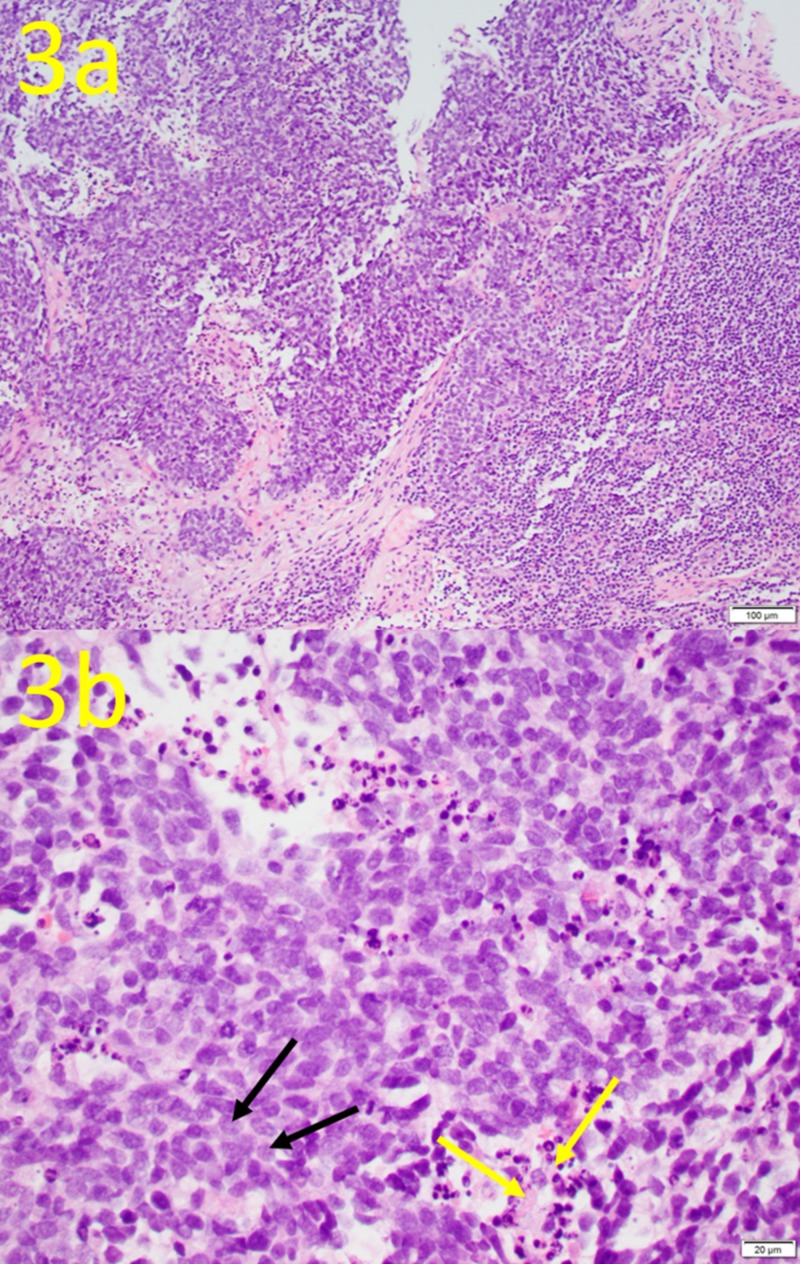
Hematoxylin and Eosin (H&E) Stain of Biopsy Tissue This H&E stain of the tissue biopsy sites demonstrates typical histological features of small cell carcinoma, with overlapping nuclei due to scanty cytoplasm and fine chromatin (black arrows) and numerous mitoses and apoptosis (yellow arrows). (a) Magnification is 20X; (b) magnification is 40X.

**Figure 4:**
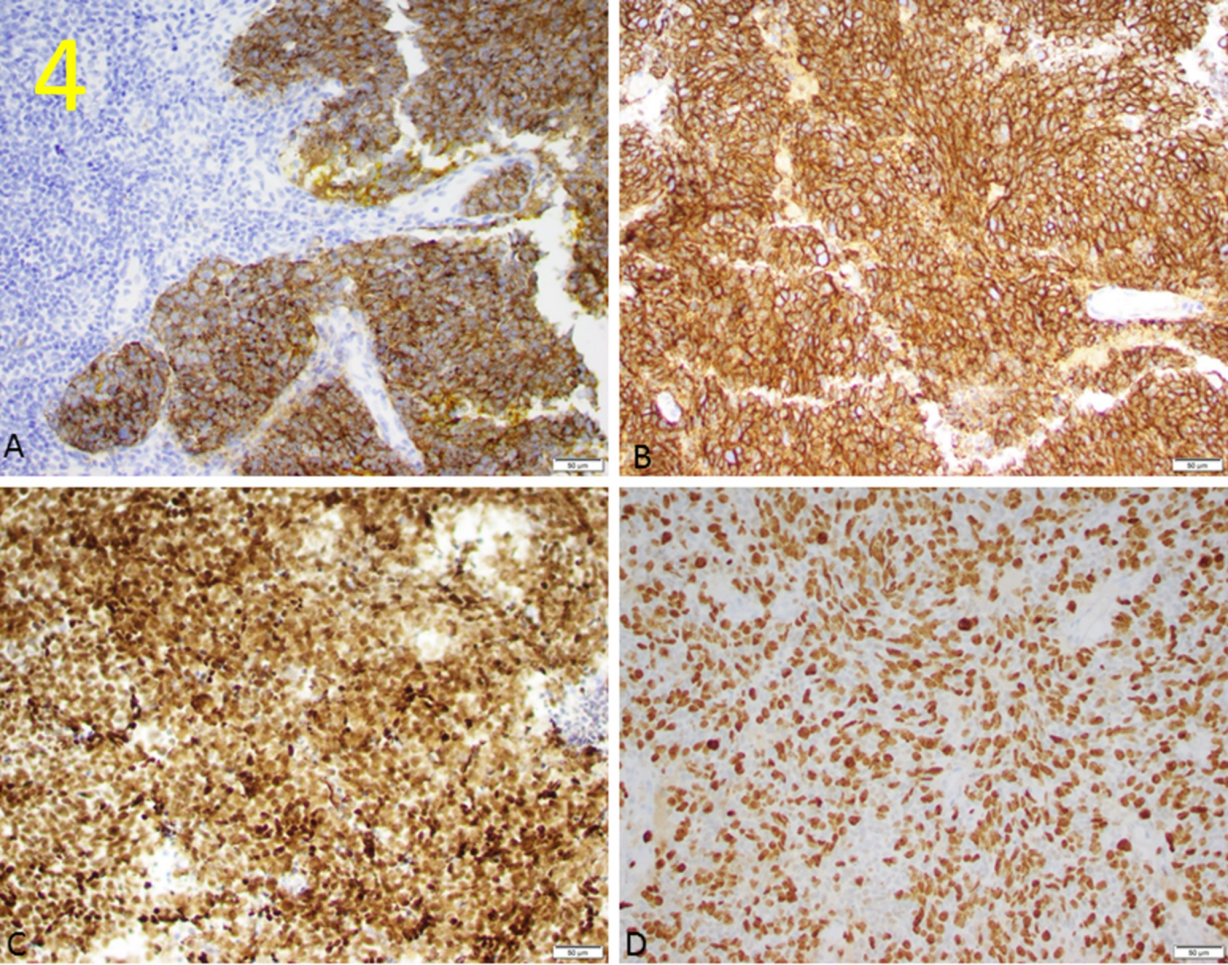
Targeted Stain of Common Neuroendocrine Carcinoma Markers These pathology images of the biopsied tissues at 20X magnification demonstrate positive staining of synaptophysin (A), TTF-1 (B), CD56 (C), and Ji-67 (D), all of which are cell markers commonly found in neuroendocrine carcinomas. Brown staining in the tissue indicates presence of the selected cell marker.

Aspiration and core needle biopsy guided by ultrasound were performed at the bilateral axilla. From the biopsy specimens, initially triple-negative metastatic invasive breast cancer was considered. Further evaluation with additional immunohistochemical studies confirms the diagnosis of bilateral metastatic SCLC, specifically poorly differentiated neuroendocrine carcinoma (Figures 3, 4).

**Figure 5:**
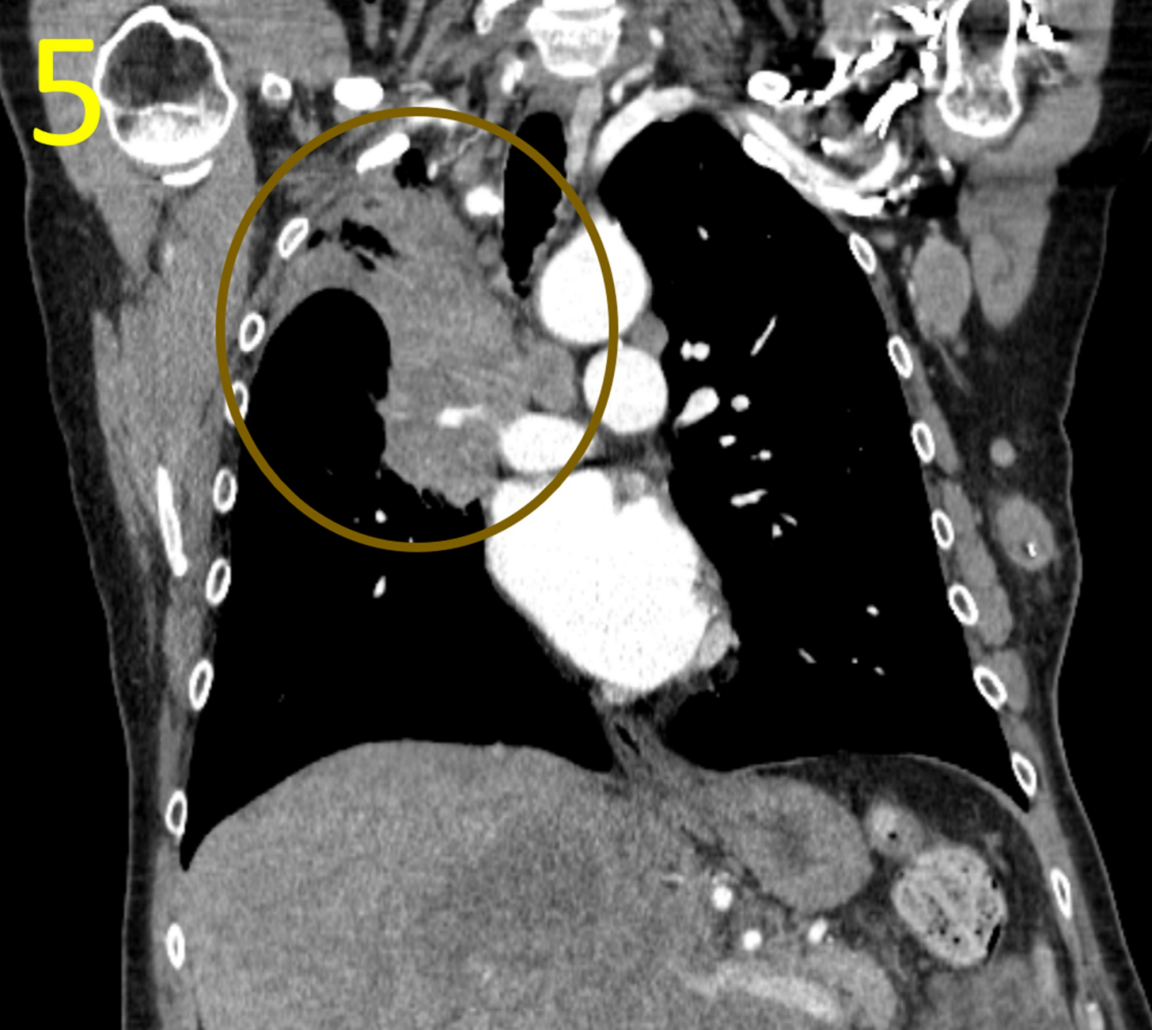
Coronal Reconstruction of Chest CT Scan This coronal reconstruction CT scan demonstrates a large right hilar mass (brown circle) causing complete collapse of right upper lobe. This mass is the likely primary site of this patient's neuroendocrine small cell lung cancer.

CT imaging was ordered to explore further possible metastasis. Chest CT scan confirmed a large, invasive right hilar mass, measuring up to 5.3 cm in the upper and middle lobes, disrupting nearby pulmonary and bronchovascular structures (Figure 5).

**Figure 6:**
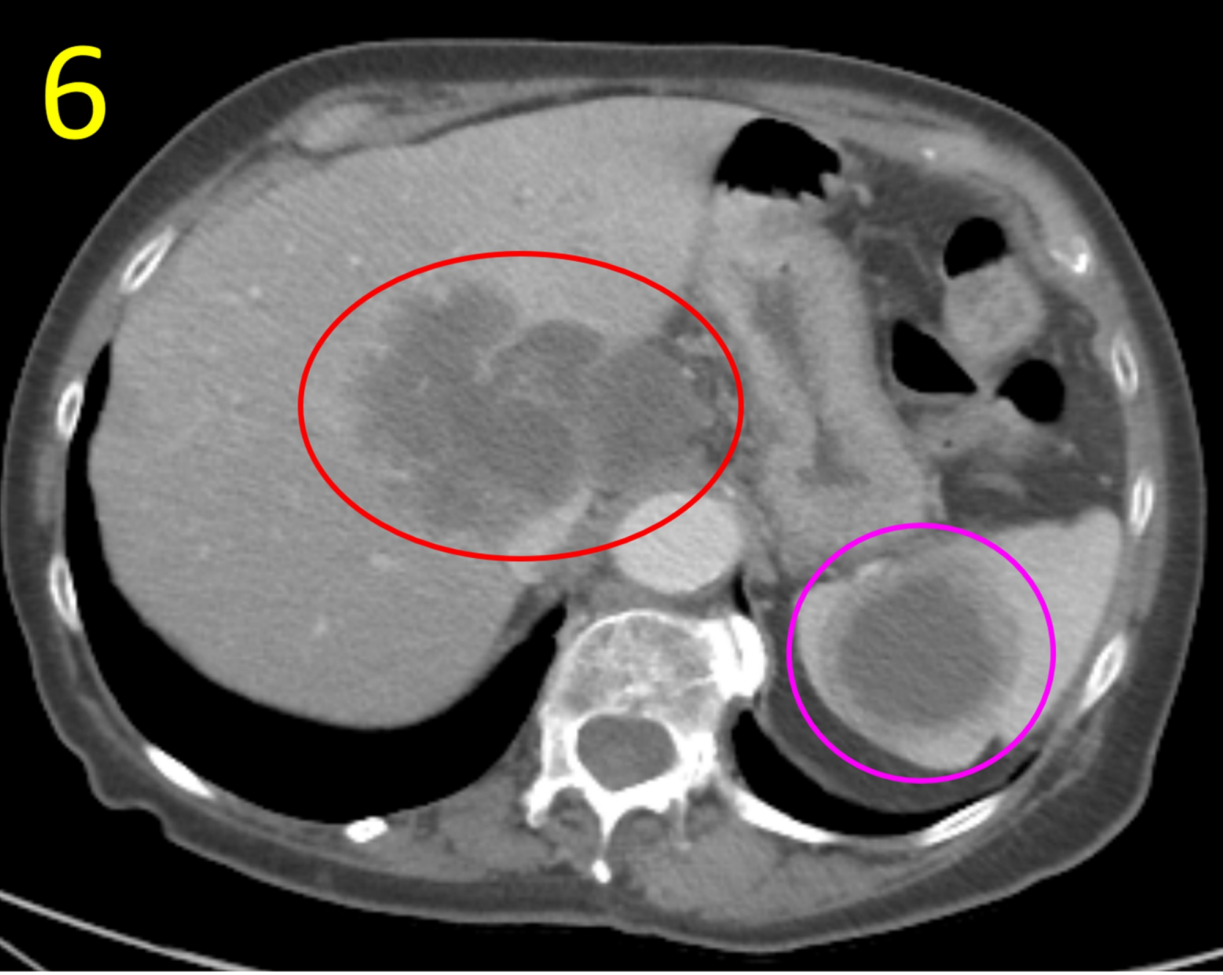
Abdominal CT Scan This abdominal CT scan demonstrates large masses in the liver (red circle) and spleen (magenta circle) that represent likely metastases from the small cell lung carcinoma mass visualized on chest CT.

The right upper lobe demonstrated complete collapse with patchy, heterogeneous consolidation, ground-glass opacity, and cortical thickening suggestive of postobstructive atelectasis or infection. Bilateral lymphadenopathy suspicious for malignancy was apparent in the axillary, mediastinal, and left hilar lymph nodes. Abdominal and pelvic CT revealed multiple lesions suspicious for metastases in the spleen and liver (Figure 6).

Additional CT of the head and MRI of the brain indicated several small areas of potential osseous or parenchymal metastases which were noted for later follow-up if there was continued concern. In combination with pathology findings from the breast biopsy, these radiological findings established a diagnosis of extensive stage SCLC. 

Upon diagnosis, the patient with high-grade neuroendocrine carcinoma was started on initial induction chemoimmunotherapy therapy for extensive stage SCLC: carboplatin-etoposide and atezolizumab, with 10 cycles planned in total. The patient has completed eight cycles of carboplatin-etoposide and atezolizumab plus two rounds of external beam radiation therapy for treatment of metastases. Sequential CT imaging has shown significant improvement in overall tumor burden over the course of several months’ treatment, with decreased size and density of masses, lymphadenopathy, and consolidation throughout the chest and abdomen. She is currently maintained on atezolizumab and is closely followed by medical oncology. Future head and brain imaging is planned to revisit suspicious cranial regions identified on initial imaging.

## Discussion

SCLC is an aggressive, high-grade neuroendocrine cancer that is most often discovered as large, obstructive lung masses in smokers. Though uncommon, cases of extrapulmonary small cell carcinoma have been documented in other organ systems, including the cervix, urinary bladder, prostate, and gastrointestinal tract [6]. Small cell carcinoma in the breast is exceptionally rare; a comprehensive literature review tallies fewer than 40 case reports [7-10]. Less than 2% of all malignancies appearing in the breast are identified as metastases from distant primary sites [11]. Of these secondary breast cancers, metastasis from SCLC is incredibly rare [12-14].

Initial imaging is often equivocal, with presentation varying widely among cases of secondary breast neuroendocrine carcinoma. In this case report, our patient’s high-grade neuroendocrine carcinoma appeared on ultrasound as complex hypoechoic axillary masses associated with loss of fatty hilum, cortical thickening, and abnormal local lymph nodes. Other case reports describe findings of calcified nodules without accompanying lymphadenopathy [11]. Accurate diagnosis relies on biopsy and cytology: small cell carcinoma classically appearing as small blue cells with poorly condensed nuclei and scant cytoplasm.

Distinguishing SCLC metastases to the breast from primary breast malignancies is an important step because of their disparate therapeutic approaches. Breast cancer treatments primarily utilize cyclin-dependent kinase inhibitors and aromatase inhibitors with adjuvant endocrine therapy. Extensively disseminated small cell carcinoma is treated similarly to SCLC, that is with a combination chemoimmunotherapy carboplatin-etoposide and atezolizumab with adjuvant targeted radiation therapy. Current literature indicates worse overall prognosis for patients with disseminated neuroendocrine carcinoma as compared to primary breast cancers [15]. From the time of original diagnosis, projected survival for patients with extensive SCLC is typically less than one year, even with treatment, and chemotherapy resistance is common. Prognosis of breast cancer depends on a wide variety of contributing factors, but good response to initial therapy may be expected to last for several years.

## Conclusions

Evaluation of newly discovered bilateral lymphadenopathy should consider autoimmune disease, infectious lymphoid hyperplasia, and solid and hematological malignancies. When this patient presented to the breast imaging department to be worked up, metastatic breast cancer led the differential. However, with further collaboration between the radiologist and pathologist, the final diagnosis was determined as neuroendocrine carcinoma. SCLC is a rare cause of breast metastases but should remain on the differential for females presenting with bilateral axillary lymphadenopathy and significant smoking history.
